# An interpolatory ansatz captures the physics of one-dimensional confined Fermi systems

**DOI:** 10.1038/srep28362

**Published:** 2016-06-21

**Authors:** M. E. S. Andersen, A. S. Dehkharghani, A. G. Volosniev, E. J. Lindgren, N. T. Zinner

**Affiliations:** 1 Department of Physics and Astronomy, Aarhus University, DK-8000 Aarhus C, Denmark; 2 Institut für Kernphysik, Technische Universität Darmstadt, 64289 Darmstadt, Germany; 3 Theoretische Natuurkunde, Vrije Universiteit Brussel, and International Solvay Institutes, Pleinlaan 2, B-1050 Brussels, Belgium; 4 Physique Théorique et Mathématique, Université Libre de Bruxelles, Campus Plaine C.P. 231, B-1050 Bruxelles, Belgium

## Abstract

Interacting one-dimensional quantum systems play a pivotal role in physics. Exact solutions can be obtained for the homogeneous case using the Bethe ansatz and bosonisation techniques. However, these approaches are not applicable when external confinement is present. Recent theoretical advances beyond the Bethe ansatz and bosonisation allow us to predict the behaviour of one-dimensional confined systems with strong short-range interactions, and new experiments with cold atomic Fermi gases have already confirmed these theories. Here we demonstrate that a simple linear combination of the strongly interacting solution with the well-known solution in the limit of vanishing interactions provides a simple and accurate description of the system for all values of the interaction strength. This indicates that one can indeed capture the physics of confined one-dimensional systems by knowledge of the limits using wave functions that are much easier to handle than the output of typical numerical approaches. We demonstrate our scheme for experimentally relevant systems with up to six particles. Moreover, we show that our method works also in the case of mixed systems of particles with different masses. This is an important feature because these systems are known to be non-integrable and thus not solvable by the Bethe ansatz technique.

Understanding the properties of low-dimensional systems is not merely an academic pursuit. Technologically promising systems such as nanotubes, nanowires, and organic conductors have one-dimensional nature^1^,^2^,^3^, while there is much evidence that high-temperature superconductors owe their spectacular properties to an effective two-dimensional structure^4^,^5^. However, in the case of interacting particles in one dimension there are still many outstanding fundamental issues in their quantum mechanical description. An avenue within which these problems can be studied is that of cold atomic gases^6^,^7^ where experiments in one-dimensional (1D) confinement can be performed with tunable interactions for systems of bosons^8^,^9^,^10^,^11^,^12^,^13^,^14^ or fermions^15^. Most recently, 1D Fermi systems have been constructed with full control over the particle number^16^ and thus engineered few-body systems are now available. This provides opportunities to study pairing, impurity physics, magnetism, and strongly interacting particles from the bottom up^17^,^18^,^19^,^20^,^21^.

The role of strong interactions in important quantum phenomena such as superconductivity and magnetism drives research into the regime of strong interactions also for 1D systems. In this respect, new theoretical approaches to confined quantum systems with strong short-range interactions have been proposed in the last couple of years^22^,^23^,^24^,^25^,^26^,^27^,^28^,^29^. Within these new developments it has become clear that the strongly interacting limit has an emergent Heisenberg spin model description. While this was realized some time ago for the case of a homogeneous system^30^, in the presence of confinement the Heisenberg model obtained has non-trivial nearest-neighbour interactions that depend on the confining potential. This can be exploited for tailoring systems to have desired static and dynamic properties^25^. In the opposite limit where we have vanishing interactions, confined 1D systems are trivially solved as the single-particle Schrödinger equation can be solved numerically to any desired level of accuracy. The natural question to ask is how to describe 1D systems with interaction strengths that are somewhere in the region between the two extreme limits?

In this paper, we propose a deceptively simple ansatz that linearly combines our knowledge of the weakly and strongly interacting limits. As an ansatz containing just two wave functions it is much easier to work with as compared to entirely numerical approaches, which typically have the solution represented on a large basis set with many non-zero contributions.

To describe the main idea we will focus on two-component Fermi systems of *N*_↑_ particles with spin projection up and *N*_↓_ particles with spin projection down. The Hamiltonian for the *N* = *N*_↑_ + *N*_↓_ system is


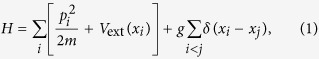


where *m* is the mass and the sums run over the number of particles, *i* = 1, …, *N*, *x*_*i*_ is the coordinate, and *p*_*i*_ the momentum of the *i*th particle. The external confinement, *V*_ext_(*x*), is assumed to be the same for all particles. Furthermore, we will assume that *V*_ext_(*x*) is parity invariant and has at least *N* bound states. The interaction strength *g* is positive for repulsive interactions and negative for attractive interactions. We note that since the parity operator commutes with the Hamiltonian, parity is conserved when *g* changes. While we only discuss the two-component Fermi system below, the case of bosons or Bose-Fermi mixtures is similar in spirit and in formalism and we return briefly to this extension in the outlook. For the majority of the discussion we assume all particles have equal mass, *m*, but we also discuss the important extension to mass-imbalanced systems. Notice that in the case of two-component Fermi systems, particles of the same spin projection will not interact due to the antisymmetry required by the Pauli principle which implies that the zero-range interaction will vanish for similar spin projections. For this reason we may use the general form of the Hamiltonian in Eq. (1) with the sum over *i* < *j* also for fermionic systems.

Consider now an *N*-body system and assume that we have solved the problem for *g* = 0 with energy eigenstate |*γ*_0_〉 and for 1/*g* = 0 with eigenstate |*γ*_∞_〉. Now form the linear combination


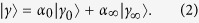


This interpolatory ansatz is motivated by the intuitive idea that the wave function of a system with intermediate-strength interactions contains a mixture of qualities from the wave functions with weak and strong interactions. We may compute 〈*γ*|*H*|*γ*〉 as a function of *α*_0_ and *α*_∞_ and look for optimum values. As we shall demonstrate in this paper, |*γ*〉 provides a simple yet accurate description of the system for any value of *g*. The only exception is deeply bound states to which we return below.

We will demonstrate that the interpolatory ansatz in Eq. (2) can capture the qualitative features of the eigenstates of the Hamiltonian in Eq. (1) and is quantitatively accurate at the level of a few percent. Furthermore, we show that the expression for the optimum energy of |*γ*〉 may be modified slightly to make it perturbatively correct in both limits of the interaction strength. With this modification, the interpolatory ansatz provides an approximation to the eigenenergy with an accuracy that is comparable to state-of-the-art numerical methods, though the ansatz is far simpler.

We provide a proof of principle by considering some important examples from the few-body limit that are experimentally relevant at the moment. For this purpose we restrict to a harmonic confinement, i.e. 
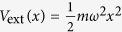
, throughout (here *ω* is the angular frequency of the oscillator).

The results we present here are:

- Analytical expressions for the ansatz parameters *α*_0_ and *α*_∞_ that depend exclusively on two matrix elements in |*γ*_0_〉 and |*γ*_∞_〉 (as well as the eigenenergies of these limiting states).
- The case *N* = 2 where one has the exact solution available^31^. Here we show that our method is accurate for both repulsive and attractive interactions to less than a few percent.
- The *N* = 3 case where no analytical solutions are known for general *g*. Here our ansatz provides very accurate results for all *g*. The results are compared to exact numerical diagonalisation utilising a unitary transformation of the interaction Hamiltonian.
- Energies for the impurity limit with *N*_↓_ = 1 and *N*_↑_ = 1–5 which we compare to experiments and find excellent agreement. We also discuss the Anderson orthogonality catastrophe for this system, which is related to the coefficient *α*_0_.
- An extension of the method to systems with particles of different mass. The examples we discuss are three-body systems and we compare to exact numerical results based on the correlated Gaussian method. This is the first application of the correlated Gaussian method to mass-imbalanced systems in 1D that we are aware of.

The ansatz we propose can be used to get very simple expressions for different observables as one needs to compute only a few matrix elements between the *g* = 0 and 1/*g* = 0 states of interest. Our method is directly extendable to bosonic systems or mixed systems, as long as one has access to the two limiting wave functions, and while we have focused on harmonically confined systems it can be straightforwardly extended to any other form of confinement. Furthermore, one may systematically improve the ansatz by adding more states.

## Results and Discussion

Let |*γ*_0_〉 be an energy eigenstate in the non-interaction limit (that is, *g* = 0) with eigenenergy *E*_0_. We now adiabatically change the interaction strength from *g* = 0 to |*g*| → ∞ and in turn we adiabatically change |*γ*_0_〉 into a new state denoted |*γ*_∞_〉 with energy *E*_∞_. The wave function of the state |*γ*_∞_〉 vanishes whenever the position coordinates of any two particles coincide, and |*γ*_∞_〉 is thus unaffected by the interaction potential


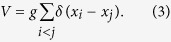


As our ansatz we now construct the trial state


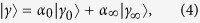


where *α*_0_ and *α*_∞_ are real parameters. Assuming |*γ*_0_〉 and |*γ*_∞_〉 are normalised, the energy of the trial state is


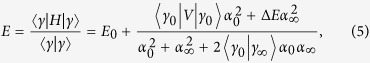


where we let Δ*E* ≡ *E*_∞_ − *E*_0_.

We use a variational approach to solving the Schrödinger equation by identifying stationary points of the trial state energy functional Eq. (5). We thus select the values of *α*_0_ and *α*_∞_ that optimise the energy of the trial state for a given value of *g*. This will yield energies and eigenstates that, although approximate, turn out to be extremely accurate as discussed below.

Before presenting the results of the variational calculation, we shall briefly examine Eq. (5) in the limiting cases of the interaction strength: If we require that *α*_∞_/*α*_0_ → 0 for *g* → 0 such that the trial state approaches |*γ*_0_〉 in the non-interacting limit, Eq. (5) gives a first-order term in *g* of the form 〈*γ*_0_|*V*|*γ*_0_〉, in agreement with first order perturbation theory. We caution, however, that requiring *α*_0_/*α*_∞_ → 0 (i.e. |*γ*〉 → |*γ*_∞_〉) for 1/*g* → 0, does not automatically ensure that the first order expansion of Eq. (5) in 1/*g* is equal to that of the exact eigenstate. We will come back to this point later on.

The coefficients that yield stationary points of Eq. (5) are given by (see the Supplementary Materials for details)


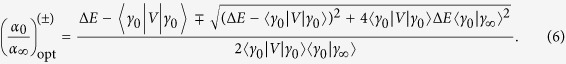


This gives the energy


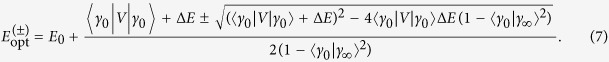


The denominator in Eq. (7) is positive because |〈*γ*_0_|*γ*_∞_〉| ≤ 1 by the Cauchy-Schwarz inequality. Hence, 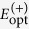 is the energy maximum while 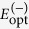 is the energy minimum. To ensure the correct energies in the limits *g* = 0 and 1/*g* = 0, we use the energy minimum, 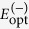, to approximate the eigenenergy whenever *g* > 0, while for *g* < 0 we use the energy maximum, 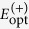.

While the interpolatory ansatz is extremely simple, it has a shortcoming in the 1/*g* → 0 limit where it does not reproduce the slope of the energy. As is shown in the following, we may, however, modify the ansatz slightly to correct this behaviour. Letting *q* ≡ −1/*g*, the first-order expansion 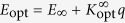 has the slope


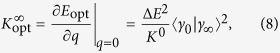


where *K*^0^ = 〈*γ*_0_|*V*|*γ*_0_〉/*g* is the corresponding slope of the energy curve in the limit of vanishing interactions. This demonstrates that the important quantity in the slope is the overlap 〈*γ*_0_|*γ*_∞_〉. In the original philosophy of the ansatz, we exploit that we know both wave functions in this overlap exactly and thus also the overlap itself; this leaves no unfixed parameters. Realising that this yields a discrepancy we have explored how to modify this assumption in order to improve the approximation.

To this end, we note that the derivation of Eq. (7) actually does not depend on |*γ*_∞_〉 being an energy eigenstate. It must be a state with energy *E*_∞_ (with respect to the non-interacting Hamiltonian), but beyond that the only requirement of |*γ*_∞_〉 is that *V*|*γ*_∞_〉 = 0. Furthermore, *E*_opt_ only depends on |*γ*_∞_〉 through the squared wave-function overlap 〈*γ*_0_|*γ*_∞_〉^2^. Thus, if we substitute 〈*γ*_0_|*γ*_∞_〉^2^ in Eqs (7 and 8) with some parameter *λ*, we may regard *E*_opt_ and 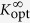 as functions of *λ*. We can then select *λ* such that *E*_opt_(*λ*) becomes perturbatively correct, that is 
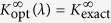
, or,


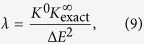


where 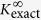 is the slope of the true eigenenergy curve at *q* = 0 (which is known exactly using the formalism of A.G.V. *et al.*^22^). We shall refer to this perturbatively correct modification, *E*_opt_(*λ*), as the *modified ansatz*. Note that this modification breaks variational bounds since we have no *a priori* knowledge of any trial state whose energy is *E*_opt_(*λ*). By going from the original interpolatory ansatz to the modified ansatz, we lose information about the wave function, but gain the correct slope of the energy at 1/*g* → 0. Below we will see that the modified ansatz increases the accuracy significantly as compared to the original ansatz.

We now proceed to discuss examples of the ansatz. Throughout our discussion, the external potential is taken to be harmonic 
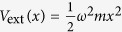
 and the same for all particles. This is the most widely studied and experimentally relevant case at the moment so it will be our focus here. Henceforth, we use natural units 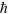 = *m* = *ω* = 1 such that energies are given in units of 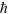
*ω*, lengths in units of 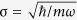, and interaction strengths *g* in units of σ
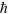
*ω*.

### Two particles

The *N* = 2 case is special as analytical results for any *g* are available due to the seminal work of Busch *et al.*^31^. It is therefore an important benchmark case for our approach. First we note that we will only be interested in the relative energy as the center of mass decouples in the harmonic trap. Note that this decoupling is not an essential assumption of our method and is merely a convenience.

**Figure 1:**
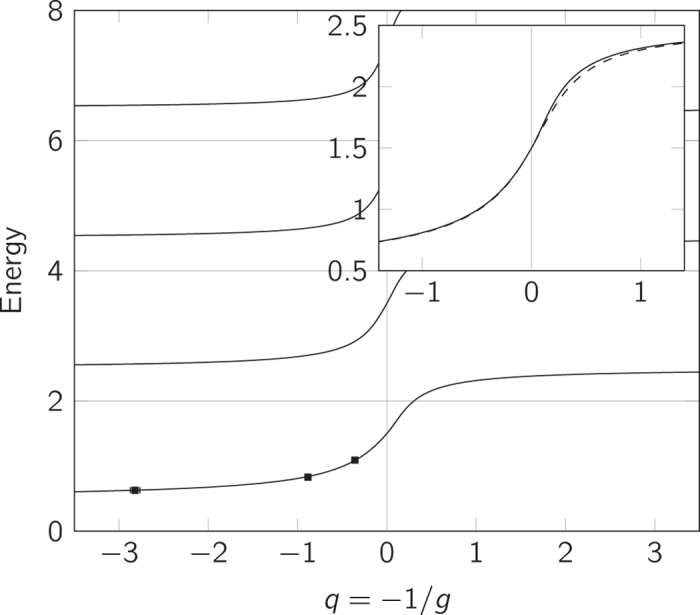
Energy spectrum for the two-particle system. Relative energy of two distinguishable fermions according to the interpolatory ansatz, showing the low-energy part of the spectrum. Only states with even parity are plotted as odd-parity states are not influenced by the zero-range interaction. The system also has a deeply bound state, but this is not plotted. Squares are experimental data points (note that the error bars are smaller than the data points)^19^. The inset is a zoom of the ground-state energy comparing the interpolatory ansatz (solid) with the exact eigenenergy (dashed) for the ground state on the repulsive side as it crosses to the attractive side.

The details on how to construct the ansatz states for the two-particle case are given in the Methods section below. The energy spectrum using the interpolatory ansatz of Eq. (7) is shown in Fig. 1. Only the even-parity solutions are shown on the plot as odd-parity states are unchanged by the zero-range interaction. The figure also includes experimental measurements of the ground-state energy. The experiment has been conducted by S. Jochim’s ultra-cold atoms group in Heidelberg, and the data originates from Wenz *et al.*^19^. This data has been corrected for the imperfections of the trap as described in Wenz *et al.*^19^. As seen, the experimental data agrees with the interpolatory ansatz well within the experimental uncertainties.

If we expand the energy in terms of *g*, the first-order term agrees with the result of ordinary non-degenerate perturbation theory. Similarly, in the limit 1/*g* = 0, the energy curve (as a function of *q*) of the true eigenstate has the same slope as that of our interpolatory ansatz.

The inset in Fig. 1 shows a zoom of the energy spectrum on the ground state and compares the energy predicted by Eq. (7) with the exact solution. For *q* > 0 the energy of the interpolatory ansatz is within 0.05 of the exact energy in the vicinity of *q* ~ 0.4 and even less elsewhere. The error decreases as we move up in the spectrum. For *q* < 0 the deviation is less than 0.006 for the ground state; again greatest around *q* ~ −0.4. On this side, the error increases for the excited states, but we find that it is bounded by about 0.03.

We conclude that the ansatz is extremely accurate for the *N* = 2 case where we can compare to analytical results^31^. One may also compare the wave functions and again find extremely good agreement (see Supplementary Fig. S1).

For attractive interactions, a deeply bound molecular state exists that we have so far ignored. However, it turns out that the ansatz of Eq. (7) can be extended to also give extremely accurate results for the deeply bound state. As is shown in the Supplementary Materials, this can be done by including an additional state in the ansatz that has the correct asymptotic behaviour as *g* → −∞. We stress that this is in fact in complete agreement with the universal philosophy of the ansatz method, i.e. interpolation between (known) extremes. Thus to address deeply bound states one needs a state in the extreme limit of large negative energy. This yields a very precise approximation also for the deeply bound state. This highlights the universal nature of our approach. We will not pursue the deeply bound states any further in this paper.

### Three particles

The simplest non-trivial example of a three-body two-component Fermi system has *N*_↑_ = 2 and *N*_↓_ = 1. The interaction potential is


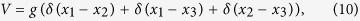


and we let *x*_1_ be the position of the spin-down fermion, while *x*_2_ and *x*_3_ denote the position of spin-up fermions. Again the third interaction term will vanish for identical fermions but we keep it for generality.

Eigenstates of the harmonic Hamiltonian are described by two quantum numbers, *ν* ≥ 0 and *μ* ≥ 1, which we shall call the radial quantum number and the angular quantum number, respectively. The eigenenergies are


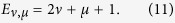


The quantum numbers *ν* and *μ* can be used to describe the energy eigenstates in both the non-interacting limit and the infinite-interaction limit.

#### Constructing the ansatz

Recall that |*γ*_0_〉 and |*γ*_∞_〉 denote eigenstates in the non-interacting limit and the infinite-interaction limit, respectively, and that we are looking for states that are adiabatically connected. As states with different radial quantum number *ν* are orthogonal, we assume that the adiabatically connected states have the same *ν* values. Parity *p* is exactly conserved and thus also the same for two states in the ansatz. The quantity that changes with *g* is therefore the *μ* quantum number, and we call the limiting values *μ*_0_ and *μ*_∞_, respectively. The angular quantum numbers are related by


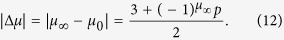


For repulsive interactions, *μ*_0_ < *μ*_∞_, and for attractive interactions, *μ*_0_ > *μ*_∞_, cf. Eq. (11). The optimum energy of the trial state can now be determined using Eq. (7) with *E*_0_ and *E*_∞_ given by Eq. (11).

#### Energy spectrum

**Figure 2:**
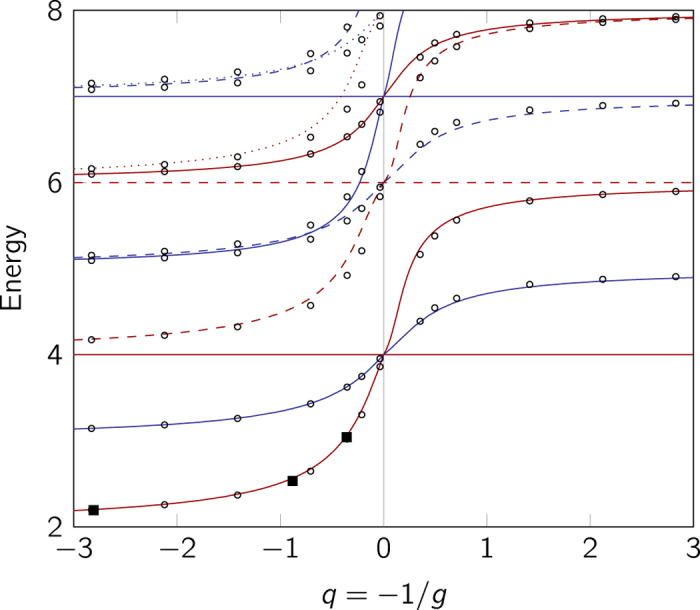
Energy spectrum of the interpolatory ansatz for the three-particle system. Red curves are energies of trial states with odd parity, and blue curves are those with even parity. Solid curves represent states with *ν* = 0, dashed curves *ν* = 1, and dotted curves *ν* = 2. Circles are numerical calculations. Deeply bound states are excluded from the plot. Squares are experimental data-points^19^. The error bars on the experimental data points are smaller than the squares and are therefore not shown.

Figure 2 shows the energy spectrum for *E* < 8 (deeply bound states are not considered) both as calculated using the interpolatory ansatz and by exact numerical diagonalisation. The interpolatory ansatz clearly describes the qualitative features of the spectrum well; both for repulsive and attractive interactions. In this energy-range, the *ν* = 0 states also give a good quantitative match to the numerical results. However, the error seems to grow with *ν*.

We see in Fig. 2 that trial states with *ν* = 0 and Δ*μ* = 1 offer a particularly good approximation to the corresponding eigenstates in the repulsive region (see e.g., the first excited state). This is because the slope of the energy curve, that is 
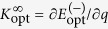
, at *q* = 0 is the same as that of the exact eigenenergy. For the trial states with Δ*μ* = 2, this is generally not the case. This result suggests that 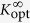 is an important quantity to reproduce correctly in an attempt such as the present to describe the physics of our problem through a simple ansatz. Note also, that the slope of the energy curve has a discontinuity at 1/*g* = 0, which contradicts the expectation that the states go smoothly through this region.

**Figure 3:**
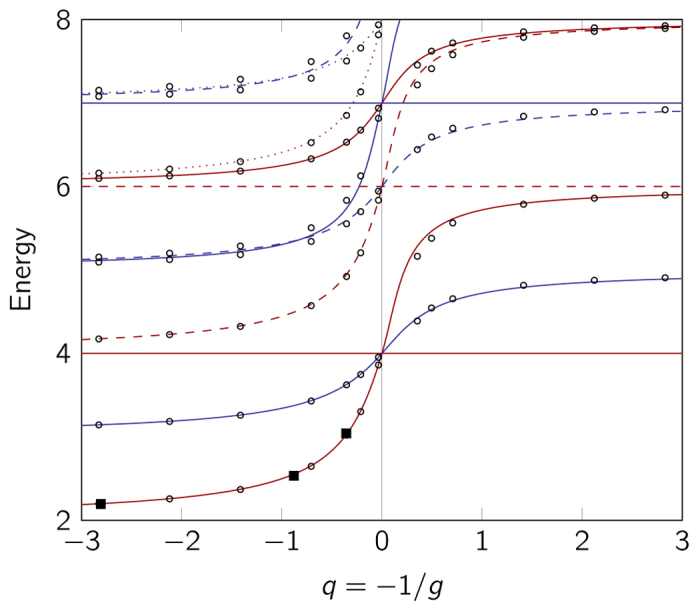
Energy spectrum for the three-particle system–modified ansatz. Energy spectrum for the three-particle system as in Fig. 2, but with the interpolatory ansatz modified to be perturbatively correct.

If we now enforce the correct slope by a modification of the interpolatory ansatz as proposed in the discussion following Eq. (8), we arrive at the spectrum shown in Fig. 3. We see that the modified ansatz agrees better with the numerical results than the original ansatz; especially for states with *ν* > 0. There are, however, still deviations on the attractive side of the spectrum, and for high energies, also on the repulsive side. We shall give a quantitative discussion of the quality of the approximation when discussing the impurity system below. We have included the experimental measurements of the ground-state energy^19^ in the figure and see that both ansatz and modified ansatz agrees very nicely with experiment, although for large *g* the modified ansatz naturally does better.

#### Mass-imbalanced systems

In the case of different masses, one typically uses another length scale given by 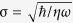 where 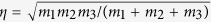, and *m*_1_ is the mass of the spin-down fermion while *m*_2_ and *m*_3_ are the masses of the spin-up fermions. We now consider the case where *m*_1_ = *M* and *m*_2_ = *m*_3_ = *m*.

**Figure 4:**
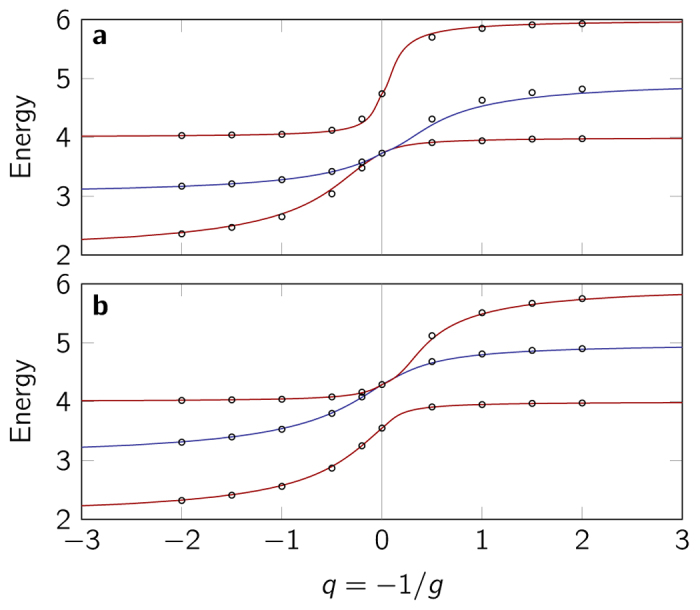
Energy spectrum for the three-particle system with different masses. Examples are given with mass ratio (**a**) *M*/*m* = 1/2 and (**b**) *M*/*m* = 2. Red curves indicate odd-parity states while blue curves indicate even-parity states. The circles are the exact results, calculated numerically using the correlated Gaussian method.

In Fig. 4 we show the energy spectrum obtained by the interpolatory ansatz for *M*/*m* = 1/2 and *M*/*m* = 2 and compare this with numerically calculated results using the correlated Gaussian approach (see the Supplementary Materials for details on the numerical methods). The agreement between the ansatz and the numerical results is striking for the low-energy part of the spectrum considered here, and we see that the ansatz can be extended also to mass-imbalanced systems.

### Impurity systems

We now consider a system of *N* fermions among which one particle (*x*_1_) is spin-down and the *N*_↑_ = *N* − 1 remaining particles (*x*_2_, …, *x*_*N*_) are spin-up. Taking into account that interactions between identical fermions vanish, we can write the interaction term as


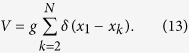


We restrict the discussion to the ground state with repulsive interactions as this has been a focus of recent experimental attention^19^. The considerations can be extended to obtain more states in the spectrum, to the attractive side, and to deeply bound states in the same manner as in the previous examples.

We denote by |*γ*_A_〉 the Slater determinant of the single-particle harmonic eigenstates 〈*x*_*k*_|*n*〉 = *ψ*_*n*_(*x*_*k*_) for *k* = 1, …, *N* and *n* = 0, …, *N* − 1. Here *ψ*_*n*_(*x*) is the single-particle eigenstate of the harmonic oscillator Hamiltonian in coordinate space with quantum number *n* and argument *x*. The state |*γ*_A_〉 is antisymmetric with respect to interchange of any two coordinates, so 〈*γ*_A_|*V*|*γ*_A_〉 = 0. We can write the antisymmetric state as


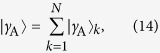


if we define


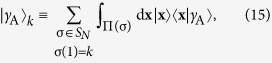


where **x** = (*x*_1_, …, *x*_*N*_), *S*_*N*_ is the symmetric group of order *N*, and Π(σ) indicates the integration region *x*_σ(1)_ <…< *x*_σ(*N*)_. In each region, Π(σ), the wave function of the ground state in the infinite-interaction limit is proportional to 〈**x**|*γ*_A_〉. The (normalised) ground state in the infinite-interaction limit is then


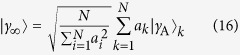


for some coefficients **a** = (*a*_1_, …, *a*_*N*_) to be determined by the method of A.G.V. *et al.*^22^ (see the Supplementary Materials). The ground state in the non-interaction limit is the single-particle harmonic eigenstate *ψ*_0_(*x*_1_) multiplied by the Slater determinant for the remaining particles in the states *ψ*_*n*_(*x*_*k*_) for *k* = 2, …, *N* and *n* = 0, …, *N* − 2.

**Figure 5:**
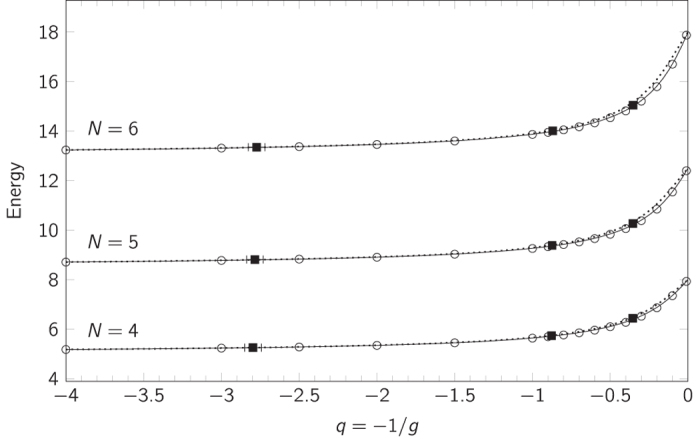
Energy spectrum of the impurity system. Energy of the ground state as predicted by the interpolatory ansatz with |*γ*_∞_〉 being the energy eigenstate in the *q* = 0 limit (dotted curve) and the modified ansatz (solid curve) for *N* = 4, 5, 6. This is compared with exact numerical calculations (circles) and experimental data (squares)^19^. Note that the error bars on the experimental data points are smaller than the squares and are only discernible for the points at small *g*.

For systems of four and five particles, the interpolatory ansatz with these |*γ*_0_〉 and |*γ*_∞_〉 yields integrals that can be evaluated analytically. For larger systems, the integrals are readily evaluated numerically. The resulting energies for *N* = 4–6 are plotted as dashed lines in Fig. 5. Here we see a very good agreement between the ansatz and the numerically exact results, and in turn excellent agreement with experimental measurements^19^. This indicates that the energetics of the system is captured by the interpolatory ansatz with high accuracy.

However, as we discussed briefly above, the ansatz does not generally reproduce the correct first-order energy term at 1/*g* = 0. Assuming that the non-interacting state should remain untouched, this prompts us to investigate whether another state can be found in the 1/*g* = 0 limit that can replace |*γ*_∞_〉 in Eq. (4) and in turn give the exact result for the slope of the energy at 1/*g* = 0. As noted above, Eq. (7) remains valid if we substitute the eigenstate |*γ*_∞_〉 with any other state with energy *E*_∞_ that obeys *V*|*γ*_∞_〉 = 0. In particular, any choice of **a** in Eq. (16) would work, and *E*_opt_ only depends on **a** through the wave-function overlap 〈*γ*_0_|*γ*_∞_〉. Hence, because *E*_opt_ is monotonic in 〈*γ*_0_|*γ*_∞_〉^2^, we may optimise the energy with respect to ***a*** for all values of *g* by maximising 〈*γ*_0_|*γ*_∞_(**a**)〉^2^ with respect to **a**. Leaving the details to the Supplementary Materials, the optimum is


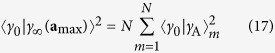


with


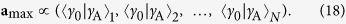


For the ground state of the *N* = 3–6 systems, however, 〈*γ*_0_|*γ*_∞_(**a**_max_)〉^2^ is very close to the known exact value of 〈*γ*_0_|*γ*_∞_〉^2^, and is not large enough to make the slope of the energy correct in the strongly-interacting limit, that is, 〈*γ*_0_|*γ*_∞_(**a**_max_)〉^2^ < *λ* with *λ* given by Eq. (9). This indicates that we cannot find a state in the infinite-interaction limit that both has the correct zeroth-order energy, *E*_∞_, and satisfies the delta-boundary conditions, *V*|*γ*_∞_〉 = 0. However, we caution that this is under the assumptions that the wave function of |*γ*_∞_〉 is continuous and has discontinuous derivatives that satisfy the boundary conditions imposed by the zero-range interaction.

We see that the interpolatory ansatz–with whatever choice of |*γ*_∞_〉–does not reproduce the correct energy slope. The modified ansatz, however, does. It is worthwhile to note that the inability to find a state, |*γ*〉 = *α*_0_|*γ*_0_〉 + *α*_∞_|*γ*_∞_〉, whose expectation value is that predicted by the modified ansatz, does not imply that the modified ansatz cannot be used. The two methods rely on different information about the system: The interpolatory ansatz requires the knowledge of the wave function at *g* = 0 and 1/*g* = 0, whereas the modified ansatz uses the knowledge of the energy behaviour. Therefore, one can use the modified anzatz to estimate the energies, and the interpolatory ansatz to approximate the wave functions.

**Figure 6:**
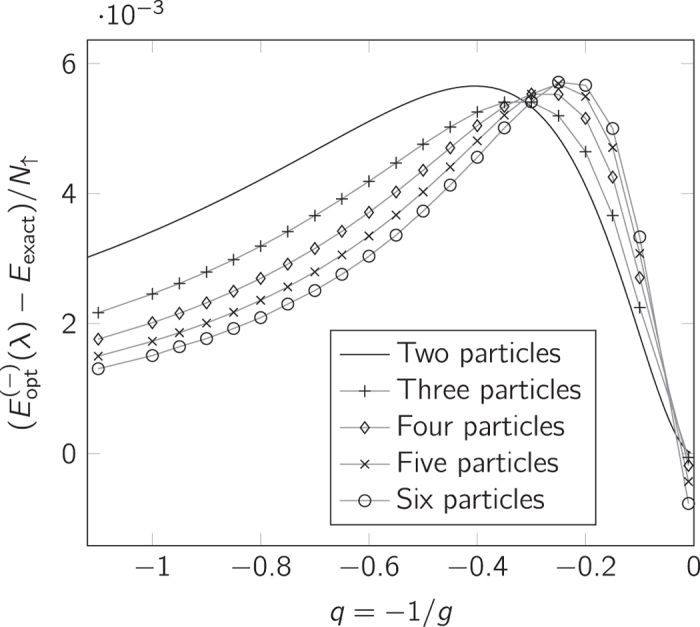
Ansatz accuracy comparison. Error in energy according to the modified ansatz compared to exact numerical results for impurity systems of *N* = 2–6 particles. (For *N* = 2 it is compared to the exact analytical solution.) For *N* ≥ 3, the gray lines between the points are a mere guide to the eye. Note the scale of the vertical axis.

Figure 5 compares the ground-state energy of the modified ansatz with results from an exact numerical diagonalisation as well as experimental data^19^. Here we see an improved agreement for large *g*. The deviations of the modified ansatz from exact numerical diagonalisation are shown in greater detail in Fig. 6. The errors are more than an order of magnitude smaller than those of the unmodified ansatz (shown in Supplementary Fig. S4).

We plot the error scaled against *N*_↑_ instead of *E*_0_ or *E*, because *E*_0_ scales as 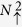 while Δ*E* = *N*_↑_. As seen in Fig. 5, the maximum in the error moves to larger interaction strength for higher *N*_↑_, i.e. with system size. There seems to be only very little increase in the magnitude of the error for larger *N*_↑_ for the system sizes we have studied. Notice that the error is slightly negative for 1/*g* ≃ 0 in the case N = 3–6. Since we know the exact energies and slopes around 1/*g* = 0, the likely cause of this is that we are pushing the accuracy of the exact numerical diagonalisation method here.

Our first observation is that the approximation of the ground-state energy offered by the modified ansatz is so good that it can compete and even in some cases beat state-of-the-art numerical exact methods. The drawback seems quite evident, i.e. we cannot be sure that any state exists in the infinite-interaction limit that when used in Eq. (5) would reproduce the energy of the modified ansatz. Thus, the modified ansatz does not immediately give us any information about the wave function in spite of its near perfect approximation of the energy. We also note that the modified ansatz breaks the variational bound on the ground state and can in principle have lower energy as compared to the exact result. However, we have clearly demonstrated that the slope of the energy at 1/*g* = 0 is an extremely important quantity for these systems as it appears crucial to reproduce in order to capture the energetics. This highlights the important role played by the recently developed theory of strongly interacting confined systems^22^.

#### Anderson overlap

Finally, we discuss the so-called Anderson overlap, which is the wave-function overlap between the non-interacting eigenstate, |*γ*_0_〉, and the interacting state, |*γ*〉, for some value of the interaction strength, *g*. This quantity is related to the Anderson orthogonality catastrophe^32^, which states that the Anderson overlap is zero in the thermodynamic limit; that is 〈*γ*_0_|*γ*〉 → 0 for *N* → ∞, and in particular 〈*γ*_0_|*γ*_∞_〉 → 0 for *N* → ∞.

**Figure 7:**
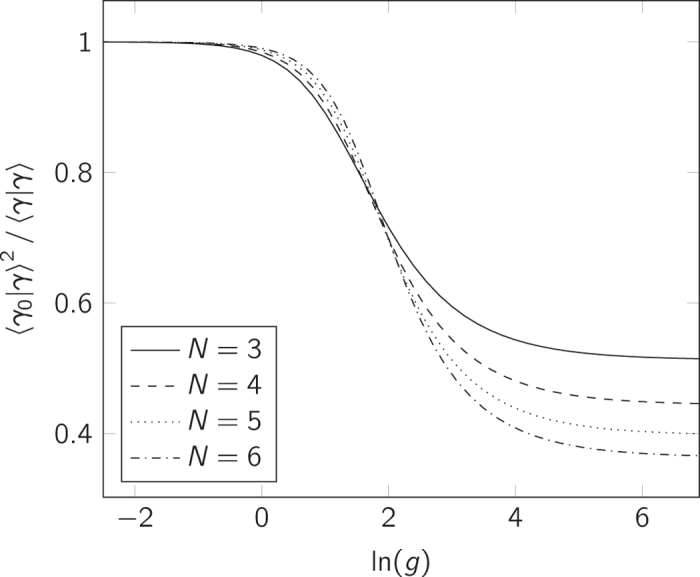
Anderson overlap according to the interpolatory ansatz. The (squared) overlap between the non-interacting energy eigenstate and the interacting state as a function of interaction strength for *N* = 3–6.

In Fig. 7 we illustrate how the overlap 〈*γ*_0_|*γ*〉^2^–with |*γ*〉 given by the interpolatory ansatz–decreases as a function of *g*. Due to the fact that we only consider a finite-sized system, the overlap does not approach zero as *g* → ∞, but the plot clearly shows that the overlap from our interpolatory ansatz tends to decrease as expected. The fact that the ansatz gives a very accurate approximation for the energy of the system does not immediately imply that this is also the case for the wave function. We leave this question for future studies, and the overlaps presented here are thus predictions based on the ansatz. It should be compared either to elaborate exact numerical calculations or to experimental measurements.

## Outlook

We have proposed a simple interpolatory ansatz for approximating the energy eigenstate of a confined, one-dimensional system of interacting particles. The ansatz is a linear combination of known eigenstates in the extreme limits of the interaction strength, *g* → 0 and 1/*g* → 0, respectively. Thanks to recent advances in the description of the eigenstates in the 1/*g* = 0 limit, both these wave functions are now available. An analytical expression for the optimum energy of this ansatz is presented which is an elementary function of only two matrix elements; the interaction energy of the eigenstate at *g* = 0, and the wave-function overlap between the eigenstates in the two limits of *g* → 0 and 1/*g* → 0. By focusing on harmonically trapped impurity systems of fermions, we have demonstrated that the ansatz is able to capture the physics of such a system. It gives us a highly accurate approximation for the energy and it also gives us a very simple expression for the wave function.

For both the two- and three-particle systems, we have been able to reproduce the entire energy spectrum with the interpolatory ansatz, save for deeply bound molecular states. The ansatz can be extended to describe deeply bound states as well; this has been shown specifically for the two-particle system. Taking the three-particle system as an example, we have also demonstrated that the ansatz works equally well for mass-imbalanced systems. A future extension of this study might investigate mass-imbalanced systems of four or more particles. It should be noted that the bottleneck here is that there are generally quite few known results about mass-imbalanced systems in the 1/*g* → 0 limit^33^. It is an open problem to find a general method that yields exact eigenstates in the strongly interacting regime for mass-imbalanced systems.

A drawback of our ansatz is that it is in general not perturbatively correct in the strongly-interacting regime. More precisely, if we take the first order derivative of the energy with respect to 1/*g* it deviates from the known exact result. We may, however, modify the expression for the energy of the interpolatory ansatz slightly such that it is perturbatively correct to linear order in *g* for *g* → 0 and to linear order in 1/*g* for 1/*g* → 0. The modified ansatz has great simplicity and accuracy at a level that is competitive with state-of-the-art numerical methods for obtaining the energy of the ground state for arbitrary *g*. Due to its simplicity it should provide a very useful tool.

We note that although the results presented here assume that the particles are trapped in an external harmonic trap, the formalism is completely general and can be applied for arbitrary external potentials with at least *N* bound single-particle states for *N*-body systems. As we have shown, the relative deviation of the energy obtained from the ansatz grows only very slowly with *N*, and there should be no problem in extending the technique to even larger systems than considered here. The decisive quantity is the overlap of the *g* = 0 and 1/*g* = 0 wave functions. This overlap may be computed using the same methods that have recently been used to compute spin chain models for strongly interacting fermions^34^ and thus scaling to larger *N* of order 30 or 40 is certainly within reach.

A future direction would be to consider a generalised version of the interpolatory ansatz where one systematically adds more states at *g* = 0 and 1/*g* = 0 in order to gradually improve the comparison. In addition, it is relatively straightforward to apply the interpolatory ansatz in the case of strongly interacting bosons^35^,^36^, or mixed systems^22^,^28^. The requirements are knowledge of states in the two limits and their overlaps so that the interpolation can be performed. The formulas for the interpolated energy given here still apply. An example could be an impurity interacting strongly with a Tonks-Girardeau gas of hard-core bosons, which is a topic of great recent interest^37^,^38^,^39^.

## Methods

In the following, we provide the details of the methods used in applying the interpolatory ansatz to two-, three- and many-particle systems.

### Details of the two-particle system

We consider a system of two distinguishable fermions and we define 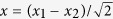. In the absence of interactions, the energy eigenstates are the harmonic eigenstates denoted |*n*〉 with integer *n* ≥ 0 (we are only concerned with the motion relative to the center of mass).

Exact solutions of the two-particle problem are available for arbitrary values of *g*^31^,^40^. The energy of an exact energy eigenstate is given indirectly by^40^


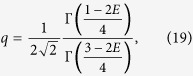


and the wave function of the state is


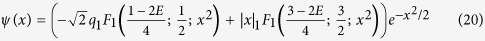


where _1_*F*_1_ is the confluent hypergeometric function of the first kind.

#### Constructing the ansatz

Let |*γ*_0_〉 = |*n*_1_〉 be an energy eigenstate in the non-interaction limit. Here |*n*_1_〉 is a single-particle eigenstate of a harmonic oscillator Hamiltonian (in the relative coordinate *x*) with quantum number *n*_1_. Furthermore, let |*γ*_∞_〉 be the corresponding eigenstate in the infinite-interaction limit–that is, |*γ*_0_〉 → |*γ*_∞_〉 as the interaction strength is changed adiabatically from *g* = 0 to 1/*g* = 0. By conservation of parity, the states |*γ*_0_〉 and |*γ*_∞_〉 have the same parity.

If |*γ*_0_〉 is odd, 〈*x* = 0|*γ*_0_〉 = 0 and thus 〈*γ*_0_|*V*|*γ*_0_〉 = 0. Hence, odd harmonic eigenstates do not change as we introduce a non-zero interaction. The even harmonic eigenstates do, however, change; the correct even eigenstate in the infinite-interaction limit is


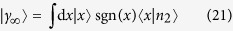


with *n*_2_ = *n*_1_ + 1 for repulsive interactions (*g* > 0) and *n*_2_ = *n*_1_ − 1 for attractive interactions (*g* < 0).

### Details of the three-particle system

Before we employ the interpolatory ansatz, we first separate out the center-of-mass motion using hyperspherical coordinates^35^,^41^. This is done merely for convenience and is not in any way essential for the approach. Defining 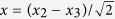 and 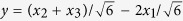, the hyperradius is given by 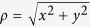 and the hyperangle is defined by tan*ϕ* = *y*/*x*. The Hamiltonian of the relative motion becomes


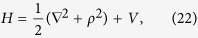


where ∇^2^ is the Laplacian in polar coordinates (*ρ*, *ϕ*) and


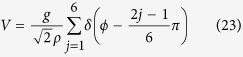


is the interaction.

#### Limiting cases

The quantum numbers *ν* and *μ* can be used to describe the energy eigenstates in both the non-interacting limit and the infinite-interaction limit. The eigenstate wave function in both limits has the general form^41^


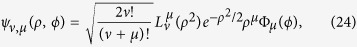


where 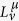 is a generalised Laguerre polynomial.

In the non-interacting limit, the (normalised) angular part of the wave function is


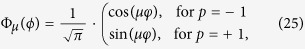


where *p* is the parity of the wave function. In this limit *p* = (−1)^*μ*^.

In the infinite-interaction limit, the wave function vanishes at the lines *ϕ* = −5*π*/6, −*π*/2, −*π*/6, *π*/6, *π*/2, 5*π*/6. In the regions between these lines, the wave function solves the Schrödinger wave equation for the harmonic oscillator without interactions.

The eigenenergies in the limit 1/*g* = 0 is given by Eq. (11) with *μ* = 3, 6, 9, 12, …. Each allowed eigenenergy is three-fold degenerate (not counting states with a different radial quantum number *ν*): One of the eigenstates in each energy triplet is a harmonic eigenstate with *μ* = 3, 6, 9, 12, … which is unaffected by the interactions. Between the two remaining eigenstates in the triplet, one is odd and the other is even.

When *μ* is even (that is, *μ* = 6, 12, 18, …) the non-trivial eigenstates in the infinite-interaction limit have the angular wave function


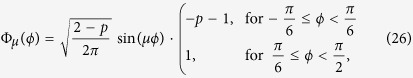


if *p* is their parity. The coefficients in the four remaining regions follow by symmetry considerations.

When *μ* is odd (*μ* = 3, 9, 15, …),


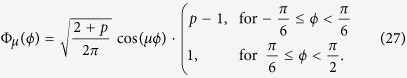


### Details of the mass-imbalanced system

The transformation into hyperspherical coordinates proceeds along the same lines with a modified Jacobian. Generally, the coordinates **x** = (*x*_1_, *x*_2_, *x*_3_) are transformed to Jacobi coordinates, **x**′ = **Jx**, through the transformation matrix


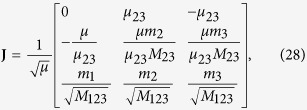


where *M*_23_ = *m*_2_ + *m*_3_, *M*_123_ = *m*_1_ + *m*_2_ + *m*_3_ and the ‘reduced’ masses are defined as 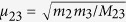 and 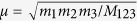. This transformation allows us to separate the center-of-mass motion from the relative motion, the solutions of the former being the well-known harmonic eigenstates. Afterwards, we transform the remaining relative coordinates into hyperspherical coordinates, *ρ* and *ϕ*, by 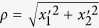 and tan(*ϕ*) = 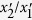.

From now on, we assume that *m*_1_ = *M* and *m*_1_ = *m*_2_ = *m*. The interaction potential can then be written as


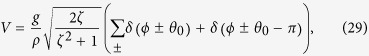


where *θ*_0_ = arctan(*ζ*) and 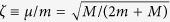. As for the equal-mass case, the energy is given by Eq. (11) 42.

The *μ* eigenvalue can be found by using parity symmetry, the Pauli principle and the delta-boundary conditions of the interaction potential. Using these conditions, one can setup an equation from which *μ* can be obtained in the limits *g* = 0 and 1/*g* = 0: For solutions with odd parity, *μ* solves the equation


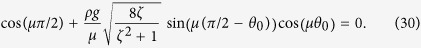


For even-parity solutions, the equation is


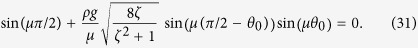


Once *μ* is found, the wave function is also known cf. Eq. (24) and the energy is given by Eq. (11) 42.

The wave function in the infinite-interaction limit now vanishes along the delta-boundary lines *ϕ* =  ± *θ*_0_, ±*π*/2, *π* ± *θ*_0_. Only when *M* = *m*, is the wave function non-zero in all six regions separated by the delta-boundary lines. In order to illustrate this, we look at two explicit examples where *M*/*m* = 1/2 and *M*/*m* = 2, respectively. For the case of *M*/*m* = 1/2, we have *θ*_0_ ≃ 0.421 (or 24.1°), and the lowest-energy solutions of Eqs (30 and 31) at 1/*g* = 0 have *μ* ≃ 0.731 (both odd and even) while the second-lowest has *μ* ≃ 3.75 (odd). The angular part of the wave function for the ground state and the first excited state in the infinite-interaction limit are


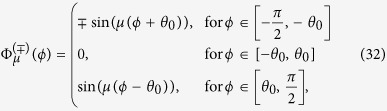


where 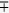 is the parity of the state. The angular part for the non-degenerate second excited state is


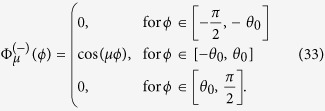


When *M*/*m* = 2, the roles are reversed. The ground state is now non-degenerate with *μ* ≃ 2.552 at *g* = ∞ and *θ*_0_ ≃ 0.615 (or 35.2°). In addition, the wave function has the same form as Eq. (33), only with different *θ*_0_ and *μ*. For the first and second excited states, the wave function has the form of Eq. (32).

One might think that there would be some continuous crossover from *M* < *m* to *M* = *m* and then to *M* > *m*, but this is not the case. Indeed, *M* = *m* is a singular case where the wave function is non-zero in all six regions.

## Additional Information

**How to cite this article**: Andersen, M. E. S. *et al.* An interpolatory ansatz captures the physics of one-dimensional confined Fermi systems. *Sci. Rep.* **6**, 28362; doi: 10.1038/srep28362 (2016).

## Supplementary Material

Supplementary Information
