# Supplementary material for: An interpolatory ansatz captures the physics of one-dimensional confined Fermi systems
Source: Sci Rep. 2016 Jun 21;6:28362. doi: 10.1038/srep28362 (PMC4914841; doi:10.1038/srep28362)
Supplement: Supplementary Information [file srep28362-s1.pdf]

An interpolatory ansatz captures the physics of one-dimensional confined Fermi systems

M. E. S. Andersen[1], A. S. Dehkharghani[1], A. G. Volosniev[2,1], E. J. Lindgren[3,4], and N. T. Zinner[1,*]

[1]Department of Physics and Astronomy, Aarhus University, DK-8000 Aarhus C, Denmark
[2]Institut für Kernphysik, Technische Universität Darmstadt, 64289 Darmstadt, Germany
[3]Theoretische Natuurkunde, Vrije Universiteit Brussel, and International Solvay Institutes, Pleinlaan 2, B-1050 Brussels, Belgium
[4]Physique Théorique et Mathématique, Université Libre de Bruxelles, Campus Plaine C.P. 231, B-1050 Bruxelles, Belgium
[*]zinner@phys.au.dk

Supplementary Materials

Stationary points of trial state energy functional

For notational convenience, we define the function

$$E'(\alpha_0, \alpha_\infty) \equiv E - E_0 = \frac{\langle \gamma_0 | V | \gamma_0 \rangle \alpha_0^2 + \Delta E \alpha_\infty^2}{\alpha_0^2 + \alpha_\infty^2 + 2\langle \gamma_0 | \gamma_\infty \rangle \alpha_0 \alpha_\infty}. \tag{S1}$$

Stationary points of E' are found where $(\partial E'/\partial \alpha_0, \partial E'/\partial \alpha_\infty) = (0,0)$. This gives the system of equations

$$\begin{bmatrix} \langle \gamma_0 | V | \gamma_0 \rangle - E' & -\langle \gamma_0 | \gamma_\infty \rangle E' \\ -\langle \gamma_0 | \gamma_\infty \rangle E' & \Delta E - E' \end{bmatrix} \begin{bmatrix} \alpha_0 \\ \alpha_\infty \end{bmatrix} = 0. \tag{S2}$$

For non-trivial solutions, the determinant of the above coefficient matrix must be zero. This yields the quadratic equation

$$0 = \left(1 - \langle \gamma_0 | \gamma_\infty \rangle^2\right) E'^2 - (\langle \gamma_0 | V | \gamma_0 \rangle + \Delta E) E' + \langle \gamma_0 | V | \gamma_0 \rangle \Delta E \tag{S3}$$

from which we arrive at Eq. (7) of the Main Text.

If a pair, $(\alpha_0, \alpha_\infty)$, realizes a stationary point of E', it solves Eq. (S2). This condition can be reduced to the relation

$$\alpha_0 = \frac{1}{\langle \gamma_0 | \gamma_\infty \rangle} \frac{E_\infty - E}{E - E_0} \alpha_\infty. \tag{S4}$$

Substituting Eq. (7) of the Main Text for E, this gives Eq. (6) of the Main Text, or equivalently, that α_0/α_∞ solves the quadratic equation

$$\langle \gamma_0 | V | \gamma_0 \rangle \langle \gamma_0 | \gamma_\infty \rangle x^2 + (\langle \gamma_0 | V | \gamma_0 \rangle - \Delta E) x - \Delta E \langle \gamma_0 | \gamma_\infty \rangle = 0. \tag{S5}$$

More on the two-particle system

Wave functions

As is shown in Fig. S1, the wave function of the ground state in the $q < 0$ region as calculated with our ansatz is very similar to the exact one[1].

Including a deeply bound state

For attractive interactions, a deeply bound molecular state exists for which the energy diverges as q approaches zero from the positive side. This state corresponds to the bound state of a delta potential well.

We may naively attempt to approximate the deeply bound state with the same trial state used for the ground state for $q < 0$; that is, Eq. (4) of the Main Text with $(n_1, n_2) = (0, 1)$. The optimised energy of this state, however, does not diverge fast enough as $g \to -\infty$. In the following, we will present a revised ansatz for the deeply bound state. Note that this is in order to improve our accuracy in the strong bound regime (large negative energy). For weaker bound states (and for repulsive interaction) the interpolatory ansatz is very accurate without additional states.

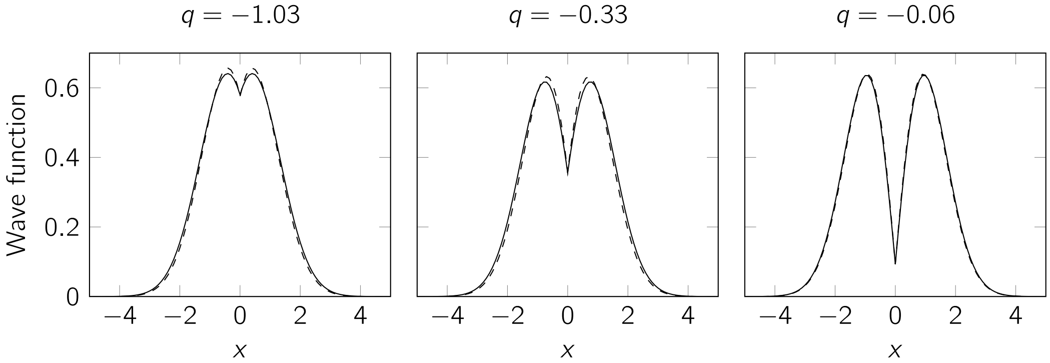

Figure S1. Wave function of the two-particle system. The ground-state wave function of the relative motion of the two particles for different values of $q = -1/g < 0$ according to the exact result (dashed) and the interpolatory ansatz (solid), respectively. The wave functions have been normalised.

For strong attractive interactions, the wave function of the deeply bound state is densely concentrated around $x = 0$ and the length scale of the harmonic trap is very large compared to the extend of the wave function. The harmonic trap is thus neglectable when $g \ll 0$, and the wave function approaches that of a delta potential well, that is,

$$\langle x|\gamma_\delta\rangle = \sqrt{\frac{|g|}{2}}\, e^{-|gx|/\sqrt{2}}, \tag{S6}$$

where we denote the ground state of the delta potential well by $|\gamma_\delta\rangle$ and its corresponding energy by $E = -\frac{1}{4}g^2$.

In this light, we may extend the ansatz with the additional state $|\gamma_\delta\rangle$:

$$|\gamma\rangle = \alpha_0|\gamma_0\rangle + \alpha_\infty|\gamma_\infty\rangle + \alpha_\delta|\gamma_\delta\rangle, \tag{S7}$$

where $|\gamma_0\rangle = |n_1 = 0\rangle$ and $|\gamma_\infty\rangle$ is given by Eq. (21) of the Main Text with $n_2 = 1$. One might be tempted to leave out the state $|\gamma_\infty\rangle$, but this state is in fact required if the trial state is to approximate the ground state not only in the limits $g \simeq 0$ and $g \ll 0$, but also in-between.

The extended ansatz gives rise to a cubic equation containing error functions, and we have solved this numerically. The resulting energy is shown in Fig. S2 and is in much better agreement with the exact energy than the original ansatz. The wave function of the extended ansatz is also very close to the exact wave function. This is evident from Fig. S3, comparing the two wave functions for three values of q.

Details of the impurity system

Denote the wave function of the n'th excited state of the single-particle harmonic oscillator ψ_n and define the Slater determinant[2]

$$F(\psi_0,\ldots,\psi_{N-1};x_1,\ldots,x_N) \equiv \frac{1}{\sqrt{N!}}\det\begin{bmatrix}\psi_0(x_1) & \psi_0(x_2) & \ldots & \psi_0(x_N)\\ \psi_1(x_1) & \psi_1(x_2) & \ldots & \psi_1(x_N)\\ \vdots & \vdots & \ddots & \vdots\\ \psi_{N-1}(x_1) & \psi_{N-1}(x_2) & \ldots & \psi_{N-1}(x_N)\end{bmatrix} \tag{S8}$$

$$= \left(\frac{2^{N-1}}{\pi}\right)^{N/4}\frac{1}{\sqrt{N!}}\left(\prod_{n=0}^{N-1}\frac{1}{\sqrt{n!}}\right)e^{-\mathbf{x}^2/2}\prod_{1\le j<k\le N}(x_k - x_j). \tag{S9}$$

The ground state in the non-interacting limit has energy $E_0 = (N_\uparrow^2+1)/2$, and with the notation defined above,

$$\langle\mathbf{x}|\gamma_0\rangle = \psi_0(x_1)F(\psi_0,\ldots,\psi_{N-2};x_2,\ldots,x_N). \tag{S10}$$

Meanwhile, the completely antisymmetric state has wave function

$$\langle\mathbf{x}|\gamma_A\rangle = F(\psi_0,\ldots,\psi_{N-1};x_1,\ldots,x_N) \tag{S11}$$

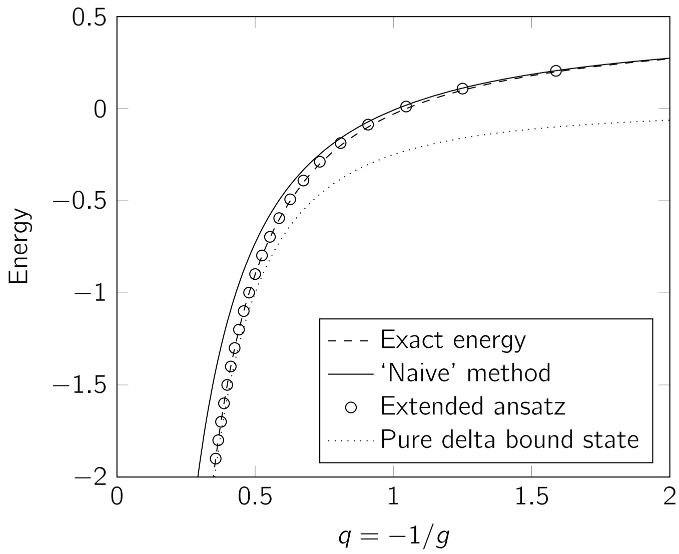

Figure S2. Energy of deeply bound state. Energy of the two-particle ground state for attractive interactions according to the exact result (dashed), the 'naive' interpolatory ansatz (solid) and the extended ansatz of Eq. (S7) (circles), respectively. For comparison, the ground-state energy of a system without the harmonic trap is also plotted (dotted).

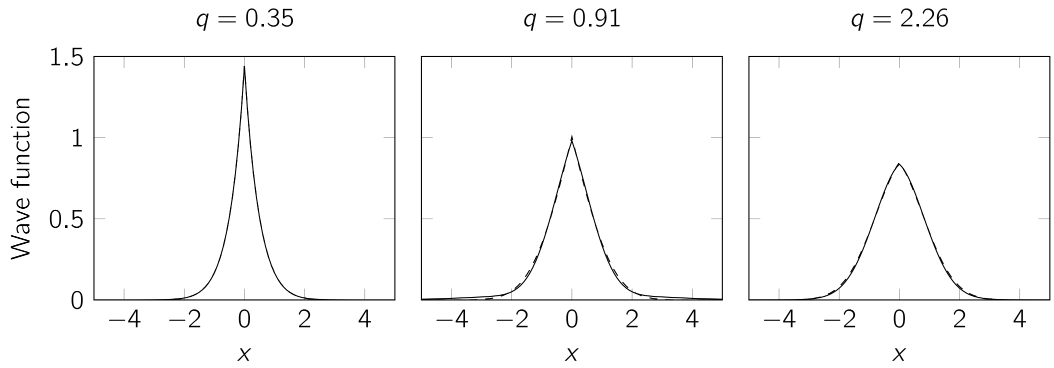

Figure S3. Wave function of deeply bound state. The two-particle ground-state wave function for different values of $q = -1/g > 0$ according to the exact result (dashed) and the extended ansatz of Eq. (S7) (solid), respectively. The wave functions have been normalised.

and energy $E_\infty = N^2/2$.

The interaction energy in the non-interacting state is

$$\langle\gamma_0|V|\gamma_0\rangle = (N-1)g\int_{-\infty}^{\infty}\mathrm{d}x_1\int_{-\infty}^{\infty}\mathrm{d}x_3\int_{-\infty}^{\infty}\mathrm{d}x_4\cdots\int_{-\infty}^{\infty}\mathrm{d}x_N\ \langle\mathbf{x}|\gamma_0\rangle^2\big|_{x_2=x_1}. \tag{S12}$$

For the ground state, this reduces to[3]

$$\langle\gamma_0|V|\gamma_0\rangle = \frac{\sqrt{2}}{\pi(N-2)!}\Gamma(N-1/2)g. \tag{S13}$$

Using Eq. (16) of the Main Text, the $\langle\gamma_0|\gamma_\infty\rangle$ may be calculated through

$$\langle\gamma_0|\gamma_\mathrm{A}\rangle_n = \binom{N-1}{n-1}\int_{-\infty}^{\infty}\mathrm{d}x_1\int_{-\infty}^{x_1}\mathrm{d}x_2\cdots\int_{-\infty}^{x_1}\mathrm{d}x_n\int_{x_1}^{\infty}\mathrm{d}x_{n+1}\cdots\int_{x_1}^{\infty}\mathrm{d}x_N\,\langle\gamma_0|\mathbf{x}\rangle\langle\mathbf{x}|\gamma_\mathrm{A}\rangle. \tag{S14}$$

Slope of energy curve

The slope of the ground-state energy curve at $g\to\infty$ is the greatest eigenvalue of the matrix[4]

$$\mathbf{A} = \begin{bmatrix} \alpha_1 & -\alpha_1 & 0 & 0 & \dots & 0 & 0 & 0 \\ -\alpha_1 & \alpha_1+\alpha_2 & -\alpha_2 & 0 & \dots & 0 & 0 & 0 \\ 0 & -\alpha_2 & \alpha_2+\alpha_3 & -\alpha_3 & \dots & 0 & 0 & 0 \\ \vdots & \vdots & \vdots & \vdots & \ddots & \vdots & \vdots & \vdots \\ 0 & 0 & 0 & 0 & \dots & -\alpha_2 & \alpha_1+\alpha_2 & -\alpha_1 \\ 0 & 0 & 0 & 0 & \dots & 0 & -\alpha_1 & \alpha_1 \end{bmatrix} \tag{S15}$$

where

$$\alpha_n = \frac{N!}{(n-1)!(N-1-n)!}\int_{-\infty}^{\infty}\mathrm{d}x_1\int_{-\infty}^{x_1}\mathrm{d}x_3\cdots\int_{-\infty}^{x_1}\mathrm{d}x_{n+1}\int_{x_1}^{\infty}\mathrm{d}x_{n+2}\cdots\int_{x_1}^{\infty}\mathrm{d}x_N\left(\frac{\langle\mathbf{x}|\gamma_\mathrm{A}\rangle}{x_1-x_2}\bigg|_{x_2=x_1}\right)^2. \tag{S16}$$

The coefficients, a_n, in Eq. (16) of the Main Text are the components of the eigenvector of $\mathbf{A}$ corresponding to the greatest eigenvalue. Note that $\alpha_n = \alpha_{N+1-n}$, which is the origin of the bisymmetry of $\mathbf{A}$.

Error in energy of interpolatory ansatz

Figure S4 shows the error in the energy of the unmodifed interpolatory ansatz compared to exact numerical methods.

Optimum wave-function overlap

By Eq. (16) of the Main Text, the squared overlap is given by

$$\langle\gamma_0|\gamma_\infty(\mathbf{a})\rangle^2 = \frac{N}{\sum_{k=1}^{N}a_k^2}\left(\sum_{n=1}^{N}a_n\langle\gamma_0|\gamma_\mathrm{A}\rangle_n\right)^2. \tag{S17}$$

Differentiating this with respect to a coefficient, a_m, and setting the result equal to zero, we get

$$0 = N\langle\gamma_0|\gamma_\mathrm{A}\rangle_m\sum_{n=1}^{N}a_n\langle\gamma_0|\gamma_\mathrm{A}\rangle_n - a_m\langle\gamma_0|\gamma_\infty(\mathbf{a}_\mathrm{max})\rangle^2, \tag{S18}$$

which is valid for $m=1,\dots,N$. Thus, we arrive at the matrix equation

$$\left(N\Big[\langle\gamma_0|\gamma_\mathrm{A}\rangle_i\langle\gamma_0|\gamma_\mathrm{A}\rangle_j\Big]_{ij} - \langle\gamma_0|\gamma_\infty(\mathbf{a}_\mathrm{max})\rangle^2\right)\mathbf{a} = 0. \tag{S19}$$

This means that $\langle\gamma_0|\gamma_\infty(\mathbf{a}_\mathrm{max})\rangle^2$ and $\mathbf{a}_\mathrm{max}$ is an eigenvalue and a corresponding eigenvector of the matrix

$$N\Big[\langle\gamma_0|\gamma_\mathrm{A}\rangle_i\langle\gamma_0|\gamma_\mathrm{A}\rangle_j\Big]_{ij} = N\begin{bmatrix}\langle\gamma_0|\gamma_\mathrm{A}\rangle_1\\ \langle\gamma_0|\gamma_\mathrm{A}\rangle_2\\ \vdots \\ \langle\gamma_0|\gamma_\mathrm{A}\rangle_N\end{bmatrix}\begin{bmatrix}\langle\gamma_0|\gamma_\mathrm{A}\rangle_1 & \langle\gamma_0|\gamma_\mathrm{A}\rangle_2 & \dots & \langle\gamma_0|\gamma_\mathrm{A}\rangle_N\end{bmatrix}. \tag{S20}$$

It is clear that the only non-zero eigenvalue belongs to the eigenvector $\mathbf{a}_\mathrm{max} = (\langle\gamma_0|\gamma_\mathrm{A}\rangle_1,\dots,\langle\gamma_0|\gamma_\mathrm{A}\rangle_N)$ and is as given in Eq. (17) of the Main Text; for there exist $N-1$ vectors orthogonal to $\mathbf{a}_\mathrm{max}$, each being an eigenvector with eigenvalue 0.

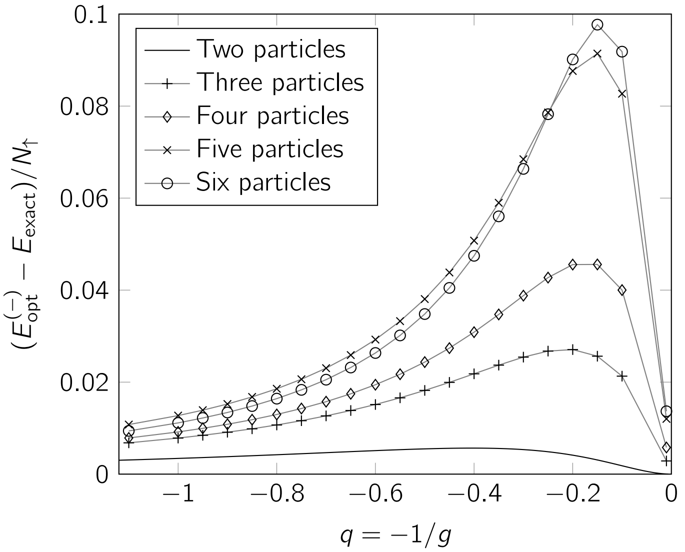

Figure S4. Accuracy of the interpolatory ansatz applied to an impurity system. Error in energy according to the unmodified interpolatory ansatz compared to exact numerical results for impurity systems of $N = 2-6$ particles. (For $N = 2$ it is compared to the exact analytical solution.) For $N \geq 3$, the gray lines between the points are a mere guide to the eye.

Numerical methods

Effective interaction approach

We consider a two-component system with N_A particles in one component and N_B particles in another, the so-called $N_A + N_B$ system. Intra-species interactions are neglected, and all the particles are assumed to have the same mass, m, and trapping frequency, ω. The general Hamiltonian of the system can then be written as:

$$\mathscr{H} = \sum_{i=1}^{N_A}\left(\frac{p_{A,i}^2}{2m} + \frac{m\omega^2}{2}q_{A,i}^2\right) + \sum_{i=1}^{N_B}\left(\frac{p_{B,i}^2}{2m} + \frac{m\omega^2}{2}q_{B,i}^2\right) + \sum_{i_A=0}^{N_A}\sum_{i_B=0}^{N_B} V_{i_A,i_B} \tag{S21}$$

where $V_{i_A,i_B} = g\delta(q_{i_A} - q_{i_B})$ are the interaction terms (g being the interaction strength), and the first two parentheses are the non-interacting part of the Hamiltonian; call it H_0. $p_{k,i}$ and $q_{k,i}$ are the momentum and coordinate operators, respectively, for particle i in subsystem $k \in \{A, B\}$. They each operate in their own subspace, so $p_{A,i} = p_i \otimes 1$ and $p_{B,i} = 1 \otimes p_i$.

The total many-body basis state is a tensor product of many-body states from each species. We refer to the states from each subsystem as few-body states and to states describing the full system as many-body states. In each subsystem we have identical fermions and therefore we need a totally antisymmetric few-body state, that is, a state that is antisymmetric under the exchange of *any two* particles:

$$|(m_1 m_2 \cdots m_N)\rangle \equiv \frac{1}{\sqrt{N!}}\sum_{\sigma\in S_N} |m_{\sigma(1)}\rangle|m_{\sigma(2)}\rangle\cdots|m_{\sigma(N)}\rangle, \tag{S22}$$

where we choose $m_1 > m_2 > \cdots > m_N$ by convention and S_N is the symmetric group of order N. The $|m_i\rangle$ represents a single-particle state equivalent to a harmonic oscillator eigenstate corresponding to eigenvalue m_i with respect to the number operator $a_i^\dagger a_i$. These single-particle states are convenient to use since they are eigenstates of H_0. The complete basis for the full system can thus be written as

$$|\Psi\rangle = |(m_1 m_2 \cdots m_{N_A})\rangle \otimes |(k_1 k_2 \cdots k_{N_B})\rangle. \tag{S23}$$

The corresponding eigenvalue to H_0 is $E = \hbar\omega(\frac{N_A+N_B}{2} + m_1 + \cdots + m_{N_A} + k_1 + \cdots + k_{N_B})$.

One of the nice properties of the Hamiltonian is that its kinetic energy and harmonic trap operators are one-particle operators, and only the interaction operator couples the particles. This means that the overall matrix is actually a sparse matrix. By construction, the contribution of the kinetic energy and harmonic trap terms are trivial. However, the interaction part is given as

$$M = \mathrm{sgn}[\sigma\tau\sigma'\tau'] V_{n_{\sigma(1)},h_{\tau(1)},n'_{\sigma'(1)},h'_{\tau'(1)}}, \tag{S24}$$

where $V_{a,b,c,d} \equiv \langle a,b|V|c,d\rangle$ is the two-body subspace matrix element and sgn is the sign function, which comes from how many times the states are swapped with each other. In addition, we are only interested in the intrinsic dynamics of states, therefore we use a Lawson projection term[5] to push away the many-body solutions corresponding to excitations of the center of mass.

An effective two-body interaction is considered instead of the bare zero-range interaction. The advantage of this effective interaction is that it converges rapidly as a function of model space size. This has been utilized to address cold atomic gases in recent papers[6,7]. It is constructed in a truncated two-body space, P, defined as the set of two-body relative harmonic oscillator states whose radial quantum numbers are smaller than a cutoff, $n_{\max}$, and it is designed such that its solutions correspond to the two-body energies that are given by the Busch formula[1]. The unitary transformation of the constructed two-body effective Hamiltonian is given as[8]

$$H_p^{\text{eff}} = \frac{U_{PP}^\dagger}{\sqrt{(U_{PP}^\dagger U_{PP})}} E_{PP}^{(2)} \frac{U_{PP}^\dagger}{\sqrt{(U_{PP}^\dagger U_{PP})}} \tag{S25}$$

where $E_{PP}^{(2)}$ is the diagonal matrix with eigenvalues from the P-space and U_{PP} is the matrix whose rows are formed by the corresponding eigenvectors. In the limit of infinite model space, $n_{\max} \to \infty$, the unitary transformation approaches the exact bare Hamiltonian results. However, the convergence to the exact limit is a lot quicker than expected for small systems. For example, $n_{\max} = 20$ for the $2+1$ system is more than enough to obtain results with a precision of 3 decimals and only a few minutes of calculation time.

Correlated Gaussian approach

Here we present the details of the correlated Gaussian approach that we have employed for the mass-imbalanced case. For further details on this method see Ref.[9]. We consider a general Hamiltonian given as

$$\mathscr{H} = \sum_{i=1}^{N_{\text{tot}}} \left(\frac{p_i^2}{2m_i} + V_{\text{ext}}(x_i) \right) + \sum_{i<j} V(x_i, x_j), \tag{S26}$$

where x_i is the coordinate of the i'th particle, $V_{\text{ext}}(x_i)$ is the external confinement and $V(x_i,x_j)$ is the 2-body interaction potential. In our case $V_{\text{ext}}(x_i) = \frac{1}{2}m\omega^2 x_i^2$ and $V(x_i,x_j) = g\delta(x_i - x_j)$, with g being the interaction strength. Please note that the specified potentials are not crucial for the method and any other system with vanishing 2-body potential for larger separation and any bounded external potential could easily do. An upper limit for the bound ground state can be found variationally based on the functional

$$E_{\text{upper}}[f] = \frac{\langle f|H|f\rangle}{\langle f|f\rangle}, \tag{S27}$$

where $|f\rangle$ is a normalizable and differentiable function built as a linear combination of states from a basis $\{\phi_k\}$: $|f\rangle = \sum_{k=1}^{l} c_k \phi_k$, where l is a computationally accessible number that is set to reach a given precision. We use a basis in the form of Gaussian functions,

$$\phi_k = e^{-(x_j - s_j^k)A_{jf}^k(x_f - s_f^k)} \equiv e^{-(\mathbf{x}-\mathbf{s})^T \mathbf{A}(\mathbf{x}-\mathbf{s}')}, \tag{S28}$$

where $\{x_j\}$ are the coordinates of the system while s_j^k and A_{jf}^k are numbers that characterize the basis elements. In order to ensure square-integrability we assume A_{jf}^k is symmetric and positive-definite. Note that Einstein's repeated summation notation is used here.

Gaussian functions usually have an analytical expression when one wants to calculate for instance $\langle \phi_k|\phi_k\rangle$ or $\langle \phi_k|\frac{\partial^2}{\partial x^2}|\phi_k\rangle$, making it very fast to calculate such expressions numerically. These functions can also be transformed easily from one Jacobi coordinate set to another and even any desirable features such as symmetry can be implemented into the ansatz.

Our next step is to choose a subset of l elements from a complete basis. This can be done in many ways, deterministically, randomly or a mix. In our calculations, we choose the first k elements with $k < l$ stochastically. This subset is the starting point of the trial function. We generate A_{jl}^k and s_j^k randomly from an appropriate distribution (e.g. exponential or Laplacian) and then determine the upper bound for the ground state. We can choose to do this step several times, say α times, and then among these α times we choose the best trial function with the lowest trial energy, call it k_{best}. Then we can start to expand k_{best} by adding some other elements randomly created, $k_{\text{best}} \mapsto k_{\text{best}} + k_{\text{add}} \leq l$ and by doing so, say β times, and then again picking the lowest energy among the β trials as our new candidate, we end up constructing a trial function that has an upper bound for the ground-state energy.

One should note, that in some situations where the functions do not decay fast enough at infinity or there are some delta functions, the number of elements in the finite basis has to be very large or go to infinity in order to describe the exact wave function everywhere. For the convergence and error of this method, see Ref.[10]. However, the method used here for our system converges relatively fast with a precision up to 4 decimals with the parameters $\alpha = 500$ and $\beta = 500$ and a calculation time of approximately 1 hour.

In order to illustrate the method, we look at our Hamiltonian for the mass-imbalanced $2+1$ system given in relative coordinates:

$$H_{\text{rel}} = T_{\text{rel}} + V_{\text{ext,rel}} + \sum_{i<j} V_{ij} \tag{S29}$$

where $T_{\text{rel}} = \frac{1}{2}(p_{x_1'}^2 + p_{x_2'}^2)$, $V_{\text{ext,rel}} = \frac{1}{2}(x_1'^2 + x_2'^2)$ and

$$\sum_{i<j} V_{ij} = \frac{g}{\hbar\omega\sigma}\left[\frac{\mu_{23}}{\sqrt{\mu}}\delta(x_1') + \frac{\sqrt{\mu}}{\mu_{23}}\delta\left(\frac{\mu}{m_1}x_1' + x_2'\right) + \frac{\sqrt{\mu}}{\mu_{23}}\delta\left(-\frac{\mu}{m_2}x_1' + x_2'\right)\right], \tag{S30}$$

with x_1', x_2', σ, μ and μ_{23} defined as in the treatment of the mass-imbalanced system in the main text. We ignore the center-of-mass Hamiltonian because its solutions are already known.

With this Hamiltonian, we can calculate the following quantities:

$$N_{ij} \equiv \langle\phi_i|\phi_j\rangle = \exp\left(-(\mathbf{s}^T\mathbf{A}\mathbf{s} + \mathbf{s}'^T\mathbf{A}'\mathbf{s}') + \frac{1}{4}\mathbf{v}^T(\mathbf{B}^{-1})^T\mathbf{v}\right)\frac{\pi^{3/2}}{\sqrt{\det(\mathbf{B})}} \tag{S31}$$

$$T_{\text{rel}} = \frac{1}{2}\langle\phi_i|\mathbf{p}^T\mathbf{p}|\phi_j\rangle = \frac{1}{2}N_{ij}\left(\text{Tr}(\mathbf{A}') - \text{Tr}(\mathbf{A}'^2\mathbf{B}^{-1}) - 2\alpha^T\mathbf{A}'^2\alpha + 4\mathbf{s}'^T\mathbf{A}'^2\alpha - 2\mathbf{s}'^T\mathbf{A}'^2\mathbf{s}'\right) \tag{S32}$$

$$V_{\text{ext,rel}} = \frac{1}{2}\langle\phi_i|\mathbf{x}_1'^T\mathbf{x}|\phi_j\rangle = \frac{1}{2}N_{ij}\left(\frac{1}{2}\text{Tr}(\mathbf{B}^{-1}) + \frac{1}{4}\mathbf{v}^T(\mathbf{B}^{-1})^2\mathbf{v}\right) \tag{S33}$$

$$\langle\phi_i|\delta(x)|\phi_j\rangle = \frac{\sqrt{\det(\mathbf{B})}}{\pi^{3/2}}N_{ij}\exp\left(-\det(\mathbf{B})\frac{\alpha_1^2}{B_{22}}\right) \tag{S34}$$

$$\langle\phi_i|\delta\left(\pm\frac{\mu}{m}x_1' + x_2'\right)|\phi_j\rangle = \frac{\sqrt{\det(\mathbf{B})}}{\pi^{3/2}}N_{ij}\frac{m}{\mu}\frac{\sqrt{\pi}}{\sqrt{B_{22} + \frac{m^2}{\mu^2}B_{11} \mp \frac{m}{\mu}2B_{12}}}\exp\left(-\det(\mathbf{B})\frac{(\alpha_1 \pm \frac{m}{\mu}\alpha_2)^2}{B_{22} + \frac{m^2}{\mu^2}B_{11} \mp \frac{m}{\mu}2B_{12}}\right), \tag{S35}$$

where $\mathbf{B} = \mathbf{A} + \mathbf{A}'$, $\mathbf{v} = 2(\mathbf{A}\mathbf{s} + \mathbf{A}'\mathbf{s}')$, $\alpha = \frac{1}{2}\mathbf{B}^{-1}\mathbf{v}$ and $m_2 = m_3 = m$. The analytical expressions for each of the integrals make it easy to calculate the value of $\langle f|H|f\rangle$ fast numerically and then try this several times for some randomly generated ansatz. In this way we can find a close upper limit to the ground-state energy of our Hamiltonian.

References

1. Busch, T., Englert, B.-G., Rzażewski, K. & Wilkens, M. Two cold atoms in a harmonic trap. *Foundations of Physics* **28**, 549–559 (1998).
2. Girardeau, M. D., Wright, E. M. & Triscari, J. M. Ground-state properties of a one-dimensional system of hard-core bosons in a harmonic trap. *Phys. Rev. A* **63**, 033601 (2001).
3. Gharashi, S. E., Yin, X. Y., Yan, Y. & Blume, D. One-dimensional fermi gas with a single impurity in a harmonic trap: Perturbative description of the upper branch. *Phys. Rev. A* **91**, 013620 (2015).
4. Volosniev, A. G., Fedorov, D. V., Jensen, A. S., Valiente, M. & Zinner, N. T. Strongly interacting confined quantum systems in one dimension. *Nat Commun* **5** (2014). Article.
5. Gloeckner, D. & Lawson, R. Spurious center-of-mass motion. *Physics Letters B* **53**, 313 – 318 (1974).

6. Christensson, J., Forssén, C., Åberg, S. & Reimann, S. M. Effective-interaction approach to the many-boson problem. *Phys. Rev. A* **79**, 012707 (2009).

7. Rotureau, J. Interaction for the trapped Fermi gas from a unitary transformation of the exact two-body spectrum. *Eur. Phys. J. D* **67**, 153 (2013).

8. Lindgren, E. J., Rotureau, J., Forssén, C., Volosniev, A. G. & Zinner, N. T. Fermionization of two-component few-fermion systems in a one-dimensional harmonic trap. *New Journal of Physics* **16**, 063003 (2014).

9. Mitroy, J. *et al.* Theory and application of explicitly correlated Gaussians. *Rev. Mod. Phys.* **85**, 693–749 (2013).

10. Volosniev, A. G. *Few-Body Systems in Low-Dimensional Geometries*. Ph.D. thesis, Aarhus University, Aarhus, Denmark (2013).
